# Integrating genetic maps in bambara groundnut [*Vigna subterranea* (L) Verdc.] and their syntenic relationships among closely related legumes

**DOI:** 10.1186/s12864-016-3393-8

**Published:** 2017-02-20

**Authors:** Wai Kuan Ho, Hui Hui Chai, Presidor Kendabie, Nariman Salih Ahmad, Jaeyres Jani, Festo Massawe, Andrzej Kilian, Sean Mayes

**Affiliations:** 1 Crops For the Future, Jalan Broga, 43500 Semenyih, Selangor Malaysia; 2 Biotechnology Research Centre, School of Biosciences, Faculty of Science, University of Nottingham Malaysia Campus, Jalan Broga, 43500 Semenyih, Selangor Malaysia; 3 School of Biosciences, Faculty of Science, University of Nottingham Sutton Bonington Campus, Sutton Bonington, Leicestershire, LE12 5RD UK; 4 Crop Science Department, Faculty of Agricultural Sciences, Sulaimani University, Sulaymaniyah, Kurdistan Region Iraq; 5 BioEasy Sdn. Bhd., Setia Alam, Seksyen U13, 40170 Shah Alam, Selangor Malaysia; 6 Diversity Arrays Technology, Bldg 3, Lv D, University of Canberra, Kirinari St., Bruce, ACT 2617 Australia

**Keywords:** Conserved synteny markers, Mapping, Genotyping-by-sequencing, Genomic comparative analysis

## Abstract

**Background:**

Bambara groundnut [*Vigna subterranea* (L) Verdc.] is an indigenous legume crop grown mainly in subsistence and small-scale agriculture in sub-Saharan Africa for its nutritious seeds and its tolerance to drought and poor soils. Given that the lack of *ex ante* sequence is often a bottleneck in marker-assisted crop breeding for minor and underutilised crops, we demonstrate the use of limited genetic information and resources developed within species, but linked to the well characterised common bean (*Phaseolus vulgaris*) genome sequence and the partially annotated closely related species; adzuki bean (*Vigna angularis*) and mung bean (*Vigna radiata*). From these comparisons we identify conserved synteny blocks corresponding to the Linkage Groups (LGs) in bambara groundnut genetic maps and evaluate the potential to identify genes in conserved syntenic locations in a sequenced genome that underlie a QTL position in the underutilised crop genome.

**Results:**

Two individual intraspecific linkage maps consisting of DArTseq markers were constructed in two bambara groundnut (2*n* = 2*x* = 22) segregating populations: 1) The genetic map of Population IA was derived from F_2_ lines (*n =* 263; IITA686 x Ankpa4) and covered 1,395.2 cM across 11 linkage groups; 2) The genetic map of Population TD was derived from F_3_ lines (*n* = 71; Tiga Nicuru x DipC) and covered 1,376.7 cM across 11 linkage groups. A total of 96 DArTseq markers from an initial pool of 142 pre-selected common markers were used. These were not only polymorphic in both populations but also each marker could be located using the unique sequence tag (at selected stringency) onto the common bean, adzuki bean and mung bean genomes, thus allowing the sequenced genomes to be used as an initial ‘pseudo’ physical map for bambara groundnut. A good correspondence was observed at the macro synteny level, particularly to the common bean genome. A test using the QTL location of an agronomic trait in one of the bambara groundnut maps allowed the corresponding flanking positions to be identified in common bean, mung bean and adzuki bean, demonstrating the possibility of identifying potential candidate genes underlying traits of interest through the conserved syntenic physical location of QTL in the well annotated genomes of closely related species.

**Conclusions:**

The approach of adding pre-selected common markers in both populations before genetic map construction has provided a translational framework for potential identification of candidate genes underlying a QTL of trait of interest in bambara groundnut by linking the positions of known genetic effects within the underutilised species to the physical maps of other well-annotated legume species, without the need for an existing whole genome sequence of the study species. Identifying the conserved synteny between underutilised species without complete genome sequences and the genomes of major crops and model species with genetic and trait data is an important step in the translation of resources and information from major crop and model species into the minor crop species. Such minor crops will be required to play an important role in future agriculture under the effects of climate change.

**Electronic supplementary material:**

The online version of this article (doi:10.1186/s12864-016-3393-8) contains supplementary material, which is available to authorized users.

## Background

Three crops account for over 60% of all food calories grown in the world; wheat (*Triticum* spp.), rice (*Oryza sativa*) and maize (*Zea mays*) with thirty crops in all accounting for around 95% of total calories consumed [1, 2]. This over-dependence on a limited number of major crops has narrowed the genetic and species base of agriculture. Growing monocultures of crop genotypes selected to respond to intensive inputs potentially makes major crops more vulnerable to pest and diseases and can lead them to perform poorly in low input systems. Exploring the potentials of underutilised and minor crops to contribute to agricultural biodiversity may also help agricultural production to cope with the effects of climate change, through improving the resilience of future agricultural systems, using crops that have been selected in-field for millennia under low input agriculture.

Underutilised and minor crops often have limited research or development funding with little or no interest from commercial seed companies. Limited resource is often a major challenge in expediting the improvement of any promising underutilised crops through marker-assisted breeding programmes. The ability to translate trait information from model and major crops species to underutilised crops is important to be able to fully exploit available resources, effectively developing research simultaneously into a complex of species, rather than a single species in isolation. Working with species complexes also allows important insights into genetic networks responsible for performance and environmental responses in the context of evolutionary relationships and ecological differences in these crops and their progenitors.

Bambara groundnut [*Vigna subterranea* (L) Verdc.] is widely grown as a plant protein source for poor farmers, particularly in sub-Saharan Africa with the seeds containing good levels of protein (18 to 26%) for human nutrition [3, 4]. The crop is drought tolerant and as a legume fixes nitrogen, it is able to tolerate low fertility soils [5] and can contribute nitrogen to agricultural systems.

Bambara groundnut is cleistogamous, highly inbreeding and has 11 pairs of chromosomes (2*n* = 2*x* = 22) [6]. The first genetic linkage map of bambara groundnut was reported by Basu in 2007 using a F_2_ segregating population (*n* = 98) derived from an interspecific cross between the non-domesticated wild type (VSSP11) and a domesticated form (DipC). An initial QTL analysis was carried out on trait differences observed for growth habit, maturity and yield production [7]. The developed map consists of 20 linkage groups and was 516 cM in length, based on 67 amplified fragment length polymorphism (AFLP) and one cross-species simple sequence repeat (SSR) marker, with the inter-marker distance varying from 4.7 to 32 cM. The first intraspecific genetic map used a F_3_ segregating population (*n* = 73) derived from a cross between two domesticated forms of bambara groundnut (Tiga Nicuru and DipC), sharing the domesticated common parental line with the interspecific cross [8]. This intraspecific map was constructed from 29 SSR and 209 DArT Array markers covering 608.6 cM in 21 linkage groups. Both parental genotypes have significant contrasting features in growth habit and seed eye pattern. Tiga Nicuru from Mali has a bunchy growth habit with longer peduncle length (*p* <0.05) and does not have eye pattern around the hilum whereas DipC collected from Botswana has a semi-spreading morphology with longer petiole and shorter internode length (*p* <0.05), greater leaf area (*p* <0.05) and has a dark eye pattern around hilum, in addition to higher pod number (*p* <0.05), seed weight per plant (*p* <0.05) and shelling percentage (*p* <0.05). A DArT Array-based UPGMA genetic distance analysis grouped the two parents into different sub-sections based on a previous population structure analysis [9], which has made these two crosses useful in unravelling our understanding of the domestication events in bambara groundnut and the genetic control of a number of morphological and physiological traits. In the future, a completed and annotated genome sequence will become available through the efforts of the African Orphan Crops Consortium (AOCC) which includes bambara groundnut as one of its targets [10]. This will greatly facilitate research in this species, but it will be some time before a fully assembled and annotated genome is available. Beyond the 101 crops identified for sequencing by AOCC, there are believed to be around 7,000 plant species which have been used by humankind, so the development of translational methodologies for the location of genetic components of traits and their underlying candidate genes is a priority for underutilised crop species. The current research presents one such generic approach, using bambara groundnut as an exemplar species.

Here we report the construction of two genetic linkage maps in two intraspecific crosses using genotyping-by-sequencing (GbS) DArTseq markers (a combination of a set of population-specific markers and a set of pre-selected common ‘link’ markers), followed by the identification of the most likely syntenic locations for the markers shared between the genomes of common bean (*Phaseolus vulgaris*), adzuki bean (*Vigna angularis*) and mung bean (*Vigna radiata*) [11–13]. These three species have 11 pairs of chromosomes and they were chosen not only because they have been sequenced and annotated (although they are at different levels of completion), but also because of the close evolutionary relationship among these legumes. The divergence time between *Phaseolus* and *Vigna* has been estimated to be 8 million years ago (MYA) [14]. The putative synthetic blocks identified across legume genomes would facilitate more effective comparison of gene order in the legume species and assist in the identification of the location of genes underlying QTL involved in controlling agronomic and yield traits in bambara groundnut, facilitating the marker-assisted selection process.

## Results and discussion

### Characterisation of DArTseq markers

35.6 and 31.1% of the total DArTseq markers generated from populations IA and TD, respectively, could be mapped to the common bean genome using the marker sequence tag, with 55% of these matches occurring in genomic contexts expected to be transcribed. In line with expectations based on genetic relatedness, a higher percentage of markers showed good sequence homology hits with the *Vigna* genomes, with more than 45% of the bambara groundnut DArTseq markers locatable on the adzuki bean and mung bean genomes (DArTseq markers from IA population: 47.8% mapped to adzuki bean genome, 46.2% to mung bean genome; DArTseq markers from TD population: 50.2% mapped to adzuki bean genome, 48.0% mapped to mung bean genome), nevertheless, some were less informative as they were found to be homologous to the remaining superscaffolds within the genome assemblies which had not been assigned to chromosome locations in the sequenced species.

### Selection of markers for genetic maps

**Fig. 1:**
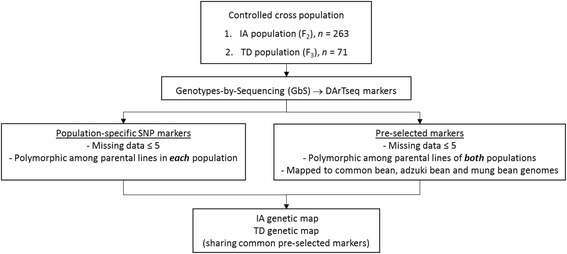
Schematic representation of our overall approach in constructing genetic maps of Population IA and TD having informative common markers from pre-selection

In our approach, two sets of DArTseq markers were used for the construction of the genetic maps; population-specific high quality SNP markers and pre-selected common ‘link’ DArTseq markers (as illustrated in Fig. 1). A total of 156 and 597 population-specific SNP markers were used for the construction of the IA map and the TD map, respectively. In addition to these high quality markers chosen using a conventional mapping approach, a set of pre-selected common DArTseq markers having the following criteria were added during map construction; 1) polymorphic in the parental lines of both populations, 2) no more than five missing genotypes, 3) sequence mappable to the common bean, adzuki bean and mung bean genomes at high stringency (as described in Method). A total of 142 markers passed these selection criteria from an unselected initial pool of 4,363 SNP markers and 7,170 dominant markers. Among these 142 pre-selected markers, 139 (97.9%) were SNP markers with the remaining 3 (2.1%) being dominant DArT markers. Of these two sets of high quality selected DArTseq markers, 268 out of 298 (89.9%) markers showed the expected segregation pattern (1:2:1 or 1:1) in the IA F_2_ segregating population and 611 out of 739 (82.7%) markers segregated in the expected pattern in the TD F_3_ population (3:2:3 or 3:5) at a significance level of *p* < 0.05.

### Genetic linkage maps from selected markers

**Fig. 2:**
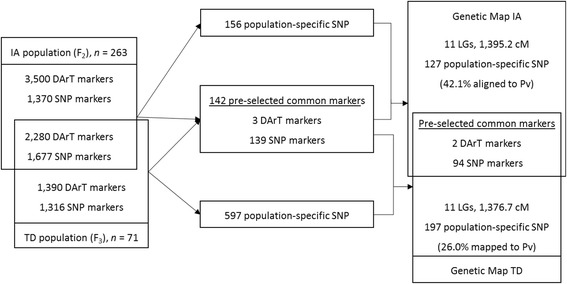
A schematic flow of the utilisation of two sets of DArTseq markers (popualtion-specific set and pre-selected common set) in both crosses for the contruction of genetic map and the result derived from this approach

**Fig. 3:**
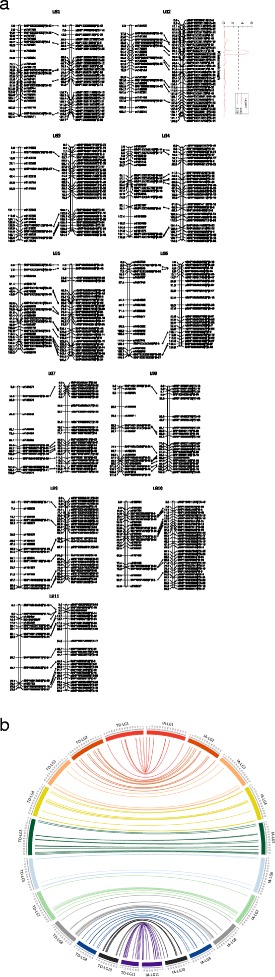
Genetic maps derived from two bambara groundnut populations; IA: F_2_ population obtained from IITA686 and Ankpa4; TD: F_3_ lines of DipC and Tiga Nicuru. Lines showing the position **(a)** and linkage **(b)** of 96 pre-selected common markers in both maps

The distribution of dominant DArT and co-dominant SNP markers across each linkage group in both populations is summarised in Table 1. Using a combination of population-specific and pre-selected markers, two framework genetic maps were constructed from the two crosses; the genetic map of IA: from 263 F_2_ lines of IITA686 x Ankpa4 gave 11 linkage groups comprising of 223 markers and covered 1,395.2 cM and the genetic map of TD: from 71 F_3_ lines of Tiga Nicuru x DipC gave 11 linkage groups consisting of 293 markers and covered 1,376.7 cM (Fig. 2). The 96 pre-selected common markers in both maps showed consistent positions within the two maps, with minor local marker order differences (generally less than 2.5 cM) probably due to the limited numbers of recombination events separating the different potential marker orders, particularly in the smaller TD cross (Fig. 3a and b) [15]. Where the orthologous marker order is consistent among the three comparison physical genomes, the chance of having a chromosomal translocation occurring within the same region of bambara groundnut genome is likely to be small, so conserved synteny can be used as a guide to marker order.

**Table 1:** The distribution of DArTseq markers (dominant DArT and co-dominant SNP) across each linkage group in both bambara groundnut populations

Linkage group	Pre-selected common markers		Population IA		Population TD	
	Dominant marker	SNP	Population-specific SNPs	Length (cM)	Population-specific SNPs	length (cM)
LG1	0	7	18	157.4	20	152.7
LG2	0	9	15	158.7	25	165.6
LG3	0	5	13	149.0	22	134.4
LG4	0	9	14	138.5	19	143.3
LG5	0	13	11	138.2	14	149.4
LG6	0	5	15	140.1	18	125.0
LG7	0	8	7	131.0	16	131.2
LG8	1	9	8	111.4	12	109.8
LG9	0	8	9	95.6	18	86.3
LG10	0	10	13	95.8	20	96.8
LG11	1	11	4	79.6	13	82.2
Total	2	94	127	1395.2	197	1376.7

### Syntenic relationships with other legume species

**Fig. 4:**
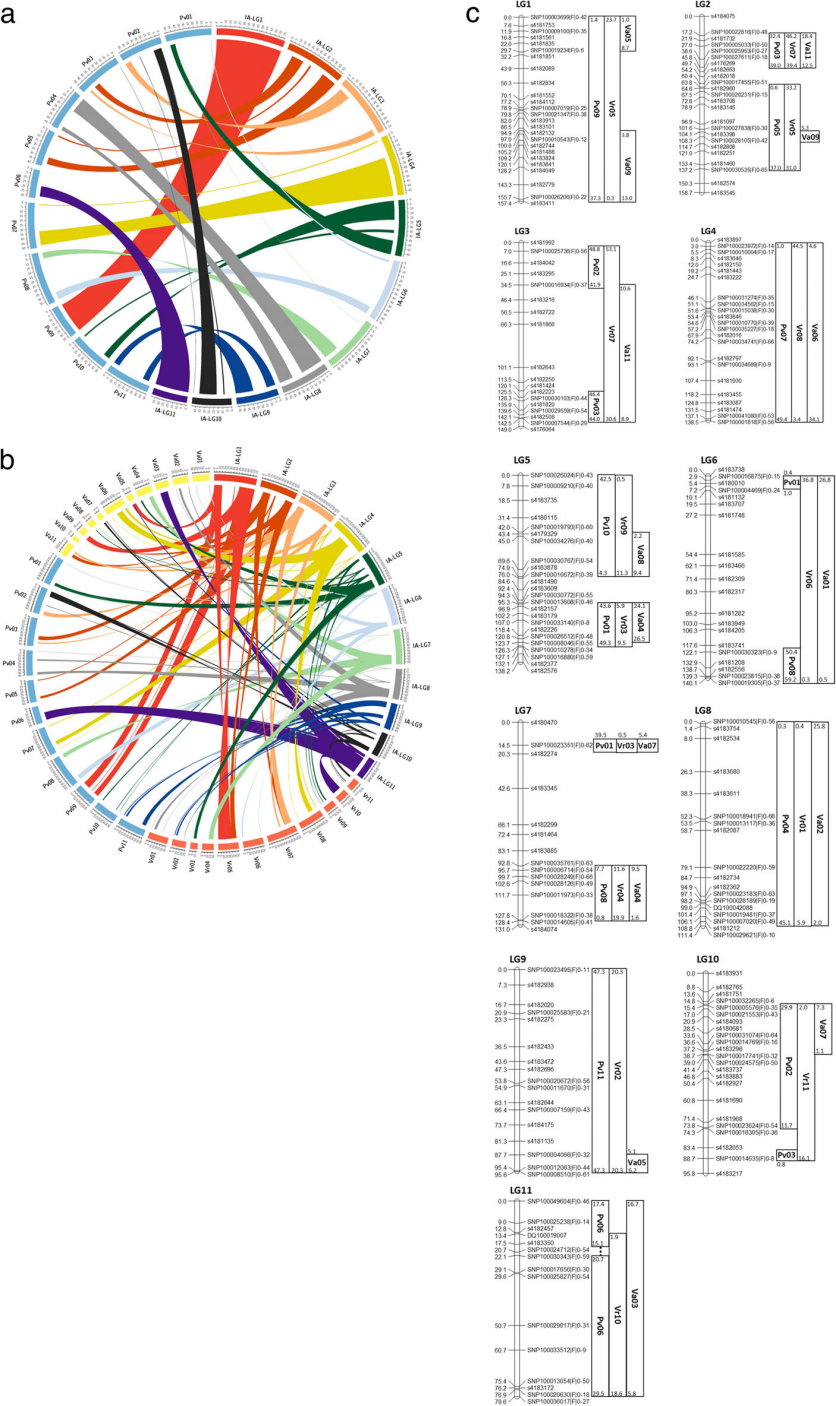
**a** This figure illustrates the conserved syntenic blocks corresponding to linkage groups of genetic Map IA in bambara groundnut compared with the common bean genome (scaling: 1 cM to 0.5 Mbp). **b** The homologous relationship of the bambara groundnut Map IA with common bean (Pv, blue), adzuki bean (Va, yellow) and mung bean (Vr, orange) genomes. The syntenic locations of the unassigned superscaffolds of aduki bean and mung bean genome have been omitted for simplicity (scaling: 1 cM to 0.5 Mbp). **c** A simplified summary of the pseudo-physical map of bambara groundnut using the syntenic locus information from common bean (Pv), adzuki bean (Va) and mung bean (Vr) across the linkage groups of bambara groundnut genetic Map IA (based on chromosome number). The mapped locations to the superscaffolds in both adzuki bean and mung bean genomes were omitted. Chromosomal location positions are in Mbp

The syntenic relationship of the bambara groundnut genetic map derived from two populations to common bean, adzuki bean and mung bean genomes is illustrated in Fig. 4a, b and c. From 11 linkage groups, four linkage groups (LG4, 8, 9 and 11 with LG9 being putative in adzuki bean genome due to a limited number of links) were found to map at the macro level to a single chromosome from each legume species. As an example, LG4 shared homology with *P. vulgaris* Chromosome 07 (Pv07), *V. radiata* Chromosome 08 (Vr08) and *V. angularis* Chromosome 06 (Va06). It is observed that the mung bean genome appears to have the highest similarity with bambara groundnut with the fewest translocations (three linkage groups), while there are seven with common bean, although, there is currently lower sequence coverage on the mung bean genome which could affect these conclusions. For example, LG3 was found to have better conserved synteny with mung bean as it corresponds to a single chromosome of mung bean and potentially adzuki bean, but two partial chromosomes of common bean. On the other hand, LG1 corresponds to Pv09 and Vr05 but two partial chromosomes of adzuki bean. A species tree constructed from the *de novo* transcriptomic assemblies of 22 accessions of 18 *Vigna* species has suggested that the divergence between bambara groundnut and other studied *Vigna* species is ~5 MYA except *V. vexillata* [12]. Our observation provides further evidence that bambara groundnut is comparatively more closely related to mung bean than to adzuki bean.

As this syntenic mapping approach is achieved through a cross-species comparison, we have been conservative in terms of mapping stringency so that the possibility of having more than one single good match location for a particular bambara groundnut marker sequence tag in the query genome was minimised. However, there is a possibility that the mapping algorithm has misidentifying the best match within the genome of the other species, or that the best match may not have been represented in the comparison species genome sequence. For example, SNP100030767 SNP |F|0-54 marker located at 69.6 cM on LG5 in Map IA has a best match position to a genomic region of Va02, whilst the adjacent markers in this linkage group mapped to Va08. A comparison of the potential sequence matching location suggested that while the Va02 position gave the higher BLAST score due to a higher percentage of matching bases (57 out of 61 bases), the second best aligned position at Va08 with a slightly lower match (56 out of 61 bases) is the better alignment and agrees with the adjacent pre-selected common markers between 0 cM and 76.0 cM of this linkage group (Additional file 1: Fig S1a and b). Therefore, for cases where the genetic location and comparative syntenic position did not match with flanking markers, we manually used BLAST to determine whether the marker was likely to be a genuine breach of conserved synteny or whether other syntenic target sites might exist. From 13 markers which do not show syntenic coherence with their neighbouring markers in either the common bean, adzuki bean or mung bean genome comparison, nine markers could be reassigned through a BLAST analysis to a syntenic location on the same chromosomes as their flanking markers. A dotted line in LG11 indicates that SNP100024712 |F|0-54 marker has the best match position on Pv07; 45,623,999 bp with one insertion, one deletion and eight mismatched bases observed but not having any good match to Pv06 as expected from its flanking markers on the same linkage group. The mapped location on Pv07 is at a genomic region flanked by AT rich sequences (496 bp and 392 bp apart upstream and downstream, respectively) and it could be mapped to the same chromosomes of adzuki bean and mung bean as the other 11 pre-selected markers on the same linkage group. Together, all these observations suggest that this locus detected a genuine divergence between *Vigna* and *Phaseolus* species.

To illustrate our approach, we have located a significant internode length QTL in the TD F_3_ population with a LOD score of 5.3 to LG2 50.6 cM (CI: 47.6 – 53.2 cM), explaining 33.4% of phenotypic variation observed in this cross (listed in Table 2 and Fig. 3a). The nearest flanking markers from the pre-selected common marker set are SNP100025953|F|0-27 and SNP100027611|F|0-18 (47.6 – 54.4 cM), which have syntenic blocks at Pv03 (38.4 – 39.1 Mbp), Va11 (12.5 – 17.4 Mbp) and Vr07 (39.4 – 43.5 Mbp). In an attempt to maximise the use of the genomic and genetic resources generated during the mung bean sequencing effort, Kang et al. identified the syntenic blocks of soybean QTL compared to the mung bean genome [12]. This has further allowed us to observe that soybean Internode Length 1–4 QTL falls within this bambara groundnut QTL conserved synteny region. This region is 1.04 Mbp in length spanning 39.8 to 40.9 Mbp of Vr07 (homologous to Gm08: 9.9 – 10.7 Mbp). The soybean internode length QTL was identified from a RIL population of Essex x Forrest (at the F_5:16_), with a LOD score of 2.7 explaining 45.7% of phenotypic variation [16]. By referring to the well-annotated common bean genome, there are a total of 49 genes underlying this corresponding regions (*Phvul.003G173800* – *Phvul.003G178600*), which could help in narrowing down the potential candidate genes for further investigation. To increase the marker resolution in this region, more population-specific markers could also be mapped to the common bean genome (as shown in Fig. 2) which would provide further information to refine the search area for candidate genes (for example, the effect of the addition of cross-species physical location information from 26% of the population-specific markers can be observed in Additional file 2: Figure S2a and b).

**Table 2:** The QTL analysis of internode length from TD F_3_ population

Traits	Cofactor	Position (cM)	Nearest marker	LOD	LIMIT	PT	PVE%	Additive effect
Internode length	nSNP100015970|F|0-21	LG2, 50.6	nSNP100015970|F|0-21 (52.2 cM)	5.3	2.1	3.1	33.4	0.65

Collectively, the finding of a predicted soybean internode length QTL (located through the mung bean genome) is in accordance with our bambara groundnut internode length QTL study and has demonstrated that our approach could be adopted in other minor and underutilised species, both to translate existing information in more studied species and to identify *de novo* candidate lists. The current coincidence of the QTL in soybean and bambara groundnut for internode length needs further investigation and the gene content within the target region between species of interest is by no means guaranteed to be the same, although the closer the species are related taxonomically, the more likely it is that they will have similar gene content in the regions of conserved synteny.

## Conclusions

The ability to use pre-selected GbS markers in two bambara groundnut crosses to generate consistent and coherent linkage maps which have conserved synteny links to the sequenced chromosomes of common bean (*Phaseolus vulgaris*), adzuki bean (*Vigna angularis*) and mung bean (*Vigna radiata*) suggests that this approach could be applied to other species of interest which have limited *ex* *ante* sequence information, but have closely related sequenced genome relatives. Our preliminary results overlaying the location of within-species QTL onto sequenced genomes suggests that translation of information (and the generation of gene lists within a trait controlling locus) can be used in an attempt to identify candidate controlling genes for further analysis within the species of interest which has no genome sequence. In bambara groundnut, before the release of whole genome sequence by AOCC, the identified potential candidate genes involved in internode length regulation could be further investigated for a better understanding of the domestication events in this species and a key trait for growth habit in different environments.

## Methods

### Mapping population

This study was based on segregation data from two populations of bambara groundnut. The F_2_ and F_3_ segregating populations were derived from a controlled cross between single genotype parental lines to produce Population IA: IITA686 (maternal) x Ankpa4 (paternal) and Population TD: Tiga Nicuru (maternal) x DipC (paternal), respectively. Plant materials were planted and trait data collected in the controlled environment FutureCrop Glasshouses, University of Nottingham Sutton Bonington Campus, UK, in 2012. The internode length trait data was recorded in mm for the average length of the fourth internode measured for the five longest stems per plant at harvest.

### DNA extraction

Young leaves were collected from 263 F_2_ individual lines, 71 F_3_ individual lines and two parental lines for each population. The DNA from the Population TD was extracted following the Dellaporta method whereas column purification method was used for Population IA (DNeasy Plant Mini Kit, Qiagen) [17]. A total of 2 μg of genomic DNA of each line were sent to DArT Pty. Ltd. (Canberra, Australia) for DArTseq genotyping.

### Marker selection for genetic map construction

DArTseq markers (as identified by the sequence tags) found in both bambara groundnut populations were mapped to common bean, adzuki bean and mung bean genomes using CLC Genomic Workbench v8 (Qiagen). The default settings for sequence mapping were used except for ‘0.8’ for ‘length fraction’ and ‘ignore’ for ‘non-specific match handling’. Subsequently, the markers mappable to all three genomes were selected further for polymorphism between all parental lines and for not more than five missing data points per individual line.

The presence or absence (0/1) scoring of dominant DArT markers for each individual line in the segregating populations were converted into genotype codes, either (c,a) or (b,d), by comparison with the parental lines. Bi-allelic SNP markers were assigned as ‘a’, ‘h’ and ‘b’ as appropriate in each individual line according to the scoring pattern in both parental lines. The markers that were filtered out in one population were also removed from the other population.

### Construction of the genetic linkage maps

A total of 142 pre-selected common markers (Additional file 3: Table S1) meeting the selection criteria were added to the population-specific SNP markers for genetic linkage analysis using JoinMap v4.1 [18]. The grouping of markers was set between LOD 2.0 and 10.0 with a step of 0.5 and the Independence LOD option adopted. All genotypic data were first analysed using a Chi-square test in JoinMap4.1 against the expected segregation patterns for the population and marker segregation type and for potential segregation distortion at a significance level of *p* <0.05. Linkage groups were established using the regression mapping approach with grouping at LOD >3.5. The Haldane mapping function with default calculation settings (recombination fraction ≤4.0, ripple value = 1, jump in goodness-of-fit threshold = 5) was selected. Each linkage group was initially screened for double crossover events. Markers showing double crossover events between two neighbouring markers within a distance of 1 to 3 cM were removed from the datasets. This reiterative process of marker removal based on graphical genotyping and ‘stress and fit’ testing allowed a high quality framework map to be generated for QTL analysis.

The physical locations of the pre-selected common DArTseq markers in the linkage maps on common bean, adzuki bean and mung bean genomes are illustrated using Circos [19].

### Detection of QTLs

Internode length trait was subjected to QTL analysis through the IM and MQM approach using MapQTL® v6.0 [20]. The analysis options were set to be default whereby the regression algorithm was used for IM and MQM mapping. The significance threshold of the LOD score was identified through permutation tests using 10,000 reiterations. The LOD score generated from IM mapping was then compared with the Genome Wide (GW) threshold at *p* ≤0.05 from the permutation test to be termed as ‘significant’. Prior to MQM mapping, the closest linked marker to the QTL with significant LOD scores was selected as a co-factor. The locations of QTLs selected by marker cofactors were verified through the LOD table and visual inspection of the LOD profile, together with a 1-LOD drop confidence interval.

## Additional files

Additional file 1: Figure S1a and **S1b.** The alignment result of SNP100030767|F|0-54 SNP marker to the different regions of adzuki bean genomes at different degrees of similarity. In this case, the second best match would be in favour as it is consistent with the syntenic physical locations of its flanking markers. (DOCX 71kb)
Additional file 2: Figure S2a and **S2b**. The syntenic relationship between linkage groups of TD population through (a) pre-selected common markers or (b) pre-selected common markers and 26% population-specific markers mappable to common bean genome (each line indicates one syntenic location data). The additional homologue information from the population-specific markers could help in further refining the target area underlying QTL. (ZIP 968kb)
Additional file 3: Table S1. Sequences of DArTseq markers in each linkage groups and their SNP positions. (XLSX 45kb)

## Abbreviations

AFLP: Amplified fragment length polymorphism
CI: Confidence interval
cM: CentiMorgan
DArT: Diversity arrays technology
GbS: Genotyping-by-sequencing
IM: Interval mapping
LG: Linkage group
LOD: Logarithm of odds
MQM: Multiple QTL mapping
PT: Permutation test
PVE: Phenotypic variation explained
QTL: Quantitative trait loci
RIL: Recombinant inbred lines
SNP: Single nucleotide polymorphism
SSR: Simple sequence repeat
UPGMA: Unweighted pair group method with arithmetic mean
