# Supplementary figures and images for: Integrating genetic maps in bambara groundnut [Vigna subterranea (L) Verdc.] and their syntenic relationships among closely related legumes
Source: BMC Genomics. 2017 Feb 20;18:192. doi: 10.1186/s12864-016-3393-8 (PMC5319112; doi:10.1186/s12864-016-3393-8)

(a)


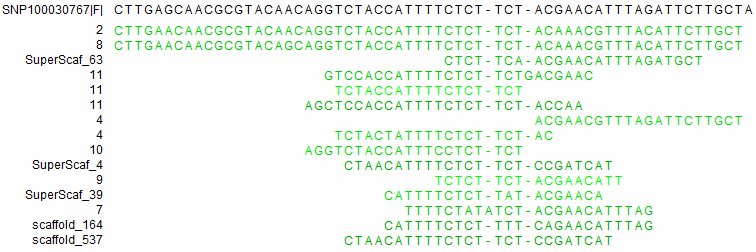


(b)


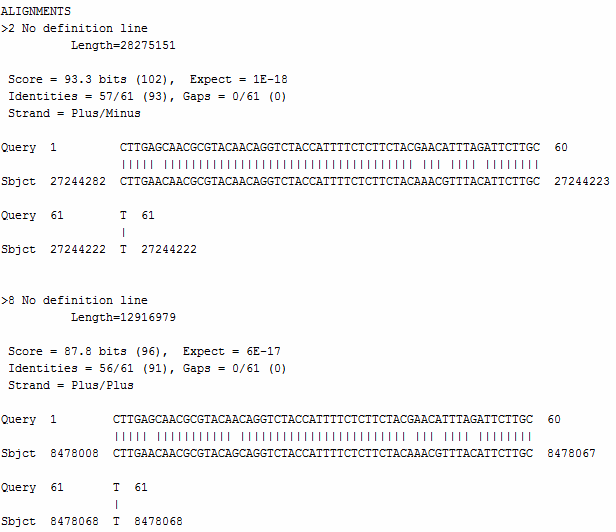

Supplement: Additional file 1: Figure S1a — and S1b. The alignment result of SNP100030767|F|0-54 SNP marker to the different regions of adzuki bean genomes at different degrees of similarity. In this case, the second best match would be in favour as it is consistent with the syntenic physical locations of its flanking markers. (DOCX 71kb) [file 12864_2016_3393_MOESM1_ESM.docx]

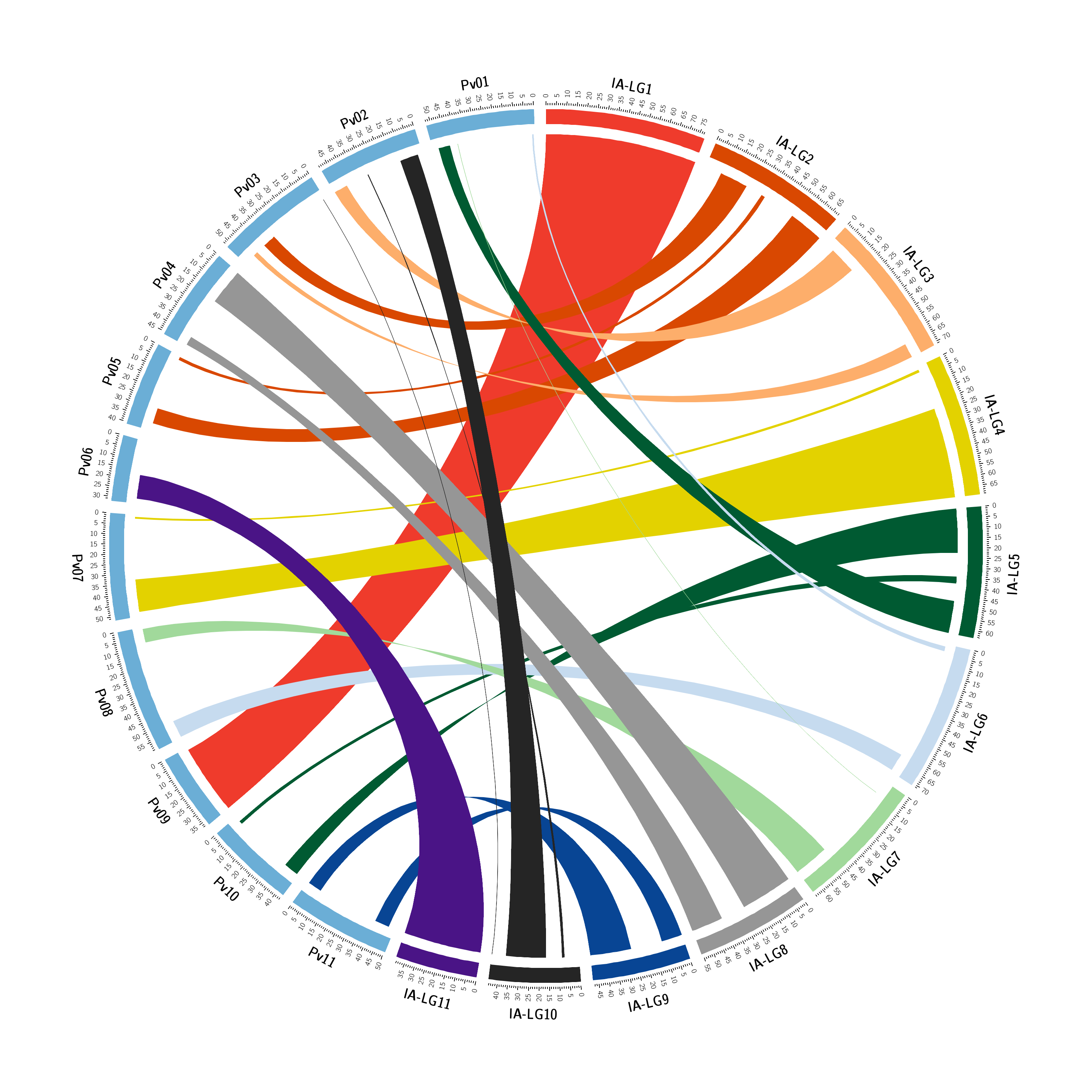

Supplement: Additional file 2: Figure S2a — and S2b. The syntenic relationship between linkage groups of TD population through (a) pre-selected common markers or (b) pre-selected common markers and 26% population-specific markers mappable to common bean genome (each line indicates one syntenic location data). The additional homologue information from the population-specific markers could help in further refining the target area underlying QTL. (ZIP 968kb) [file 12864_2016_3393_MOESM2_ESM.zip › 12864_2016_3393_MOESM2_ESM/Fig 4a.png]

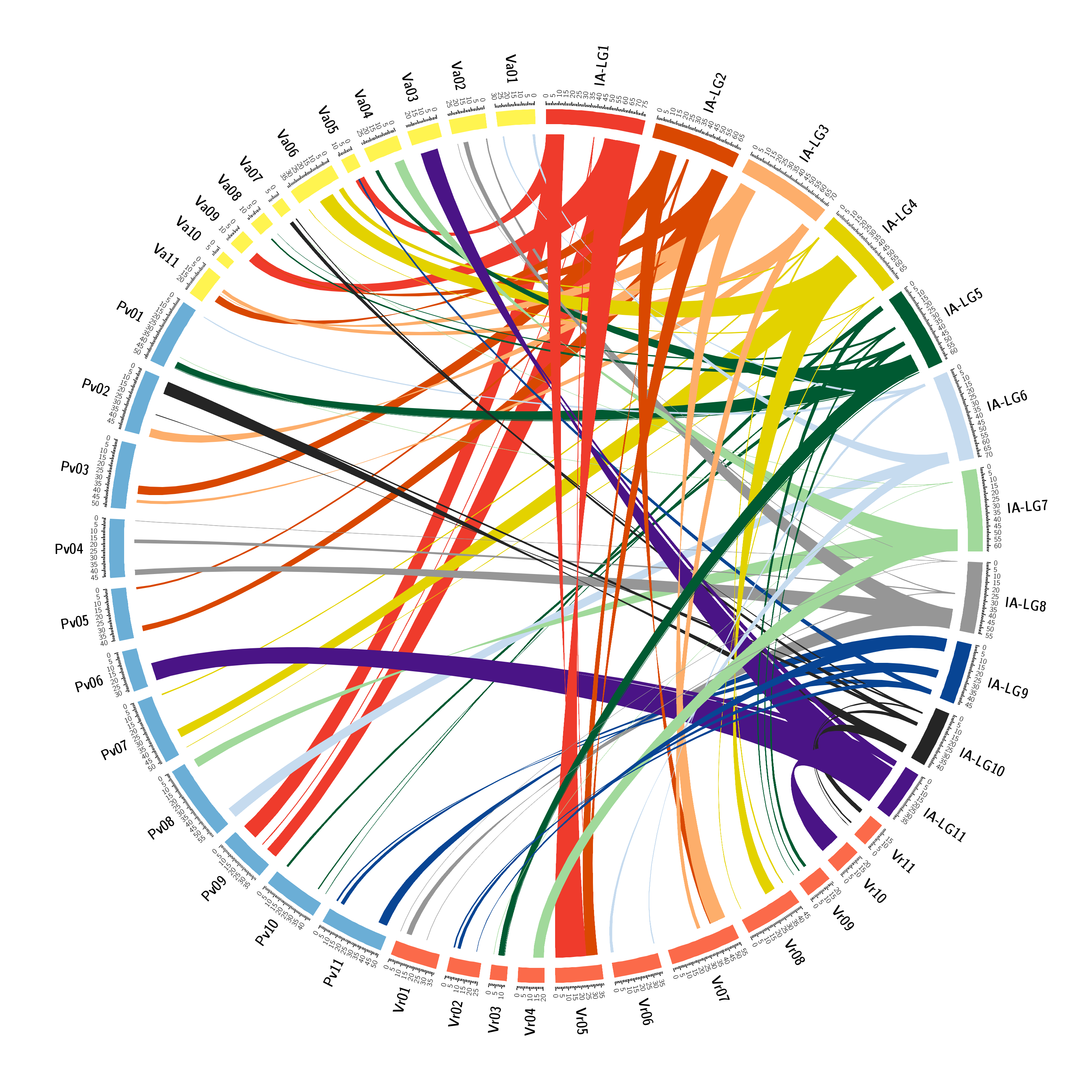

Supplement: Additional file 2: Figure S2a — and S2b. The syntenic relationship between linkage groups of TD population through (a) pre-selected common markers or (b) pre-selected common markers and 26% population-specific markers mappable to common bean genome (each line indicates one syntenic location data). The additional homologue information from the population-specific markers could help in further refining the target area underlying QTL. (ZIP 968kb) [file 12864_2016_3393_MOESM2_ESM.zip › 12864_2016_3393_MOESM2_ESM/Fig 4b.png]

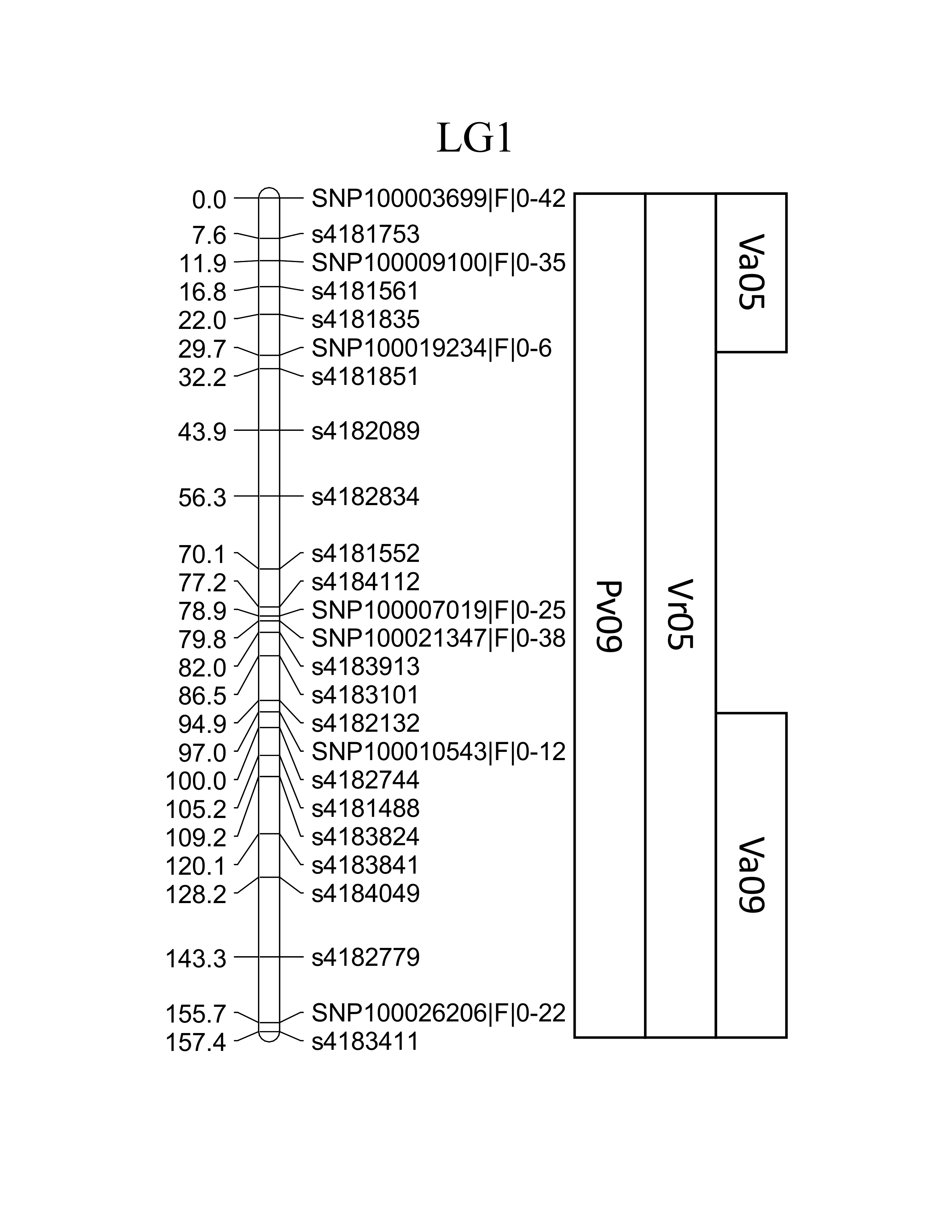

Supplement: Additional file 2: Figure S2a — and S2b. The syntenic relationship between linkage groups of TD population through (a) pre-selected common markers or (b) pre-selected common markers and 26% population-specific markers mappable to common bean genome (each line indicates one syntenic location data). The additional homologue information from the population-specific markers could help in further refining the target area underlying QTL. (ZIP 968kb) [file 12864_2016_3393_MOESM2_ESM.zip › 12864_2016_3393_MOESM2_ESM/Fig 4c/LG1.tif]

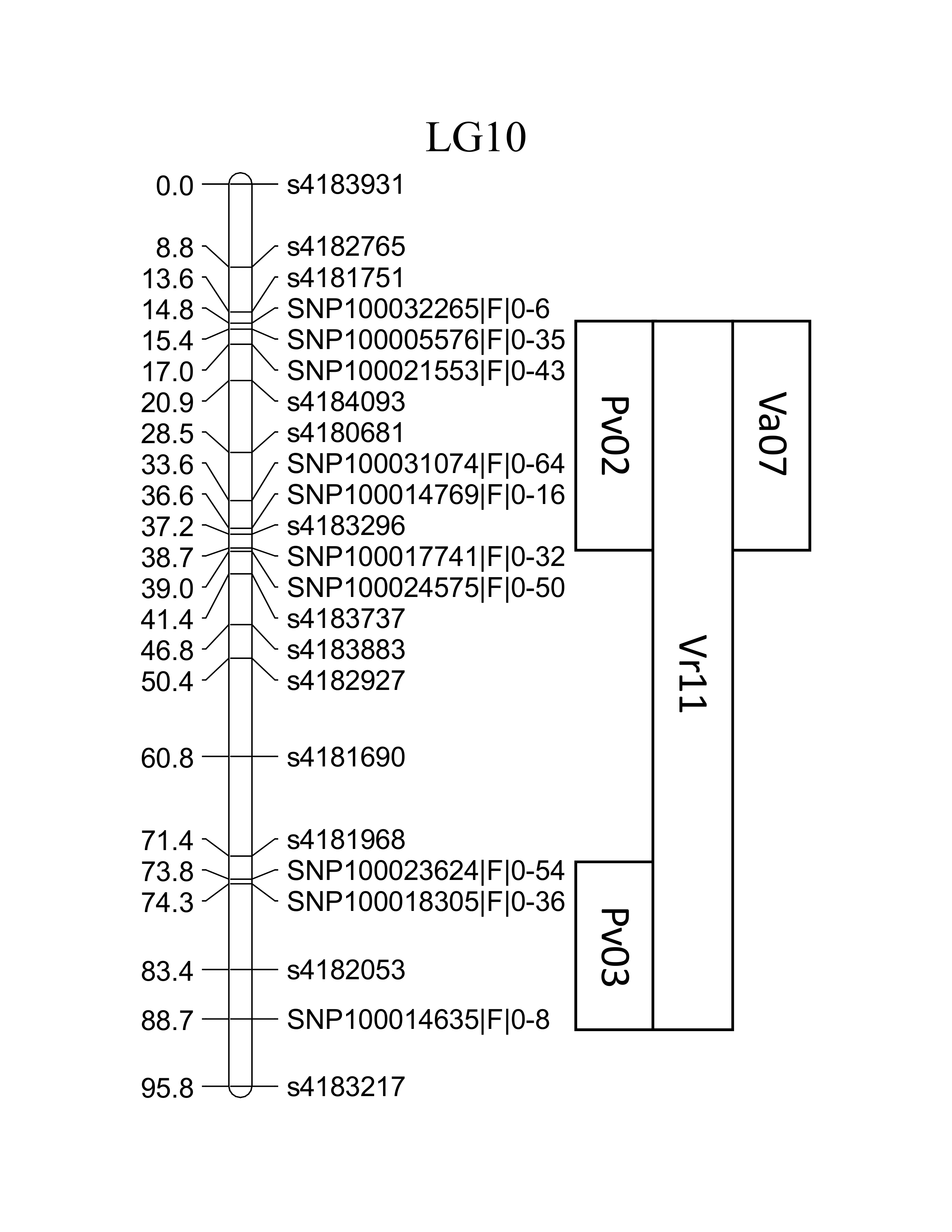

Supplement: Additional file 2: Figure S2a — and S2b. The syntenic relationship between linkage groups of TD population through (a) pre-selected common markers or (b) pre-selected common markers and 26% population-specific markers mappable to common bean genome (each line indicates one syntenic location data). The additional homologue information from the population-specific markers could help in further refining the target area underlying QTL. (ZIP 968kb) [file 12864_2016_3393_MOESM2_ESM.zip › 12864_2016_3393_MOESM2_ESM/Fig 4c/LG10.tif]

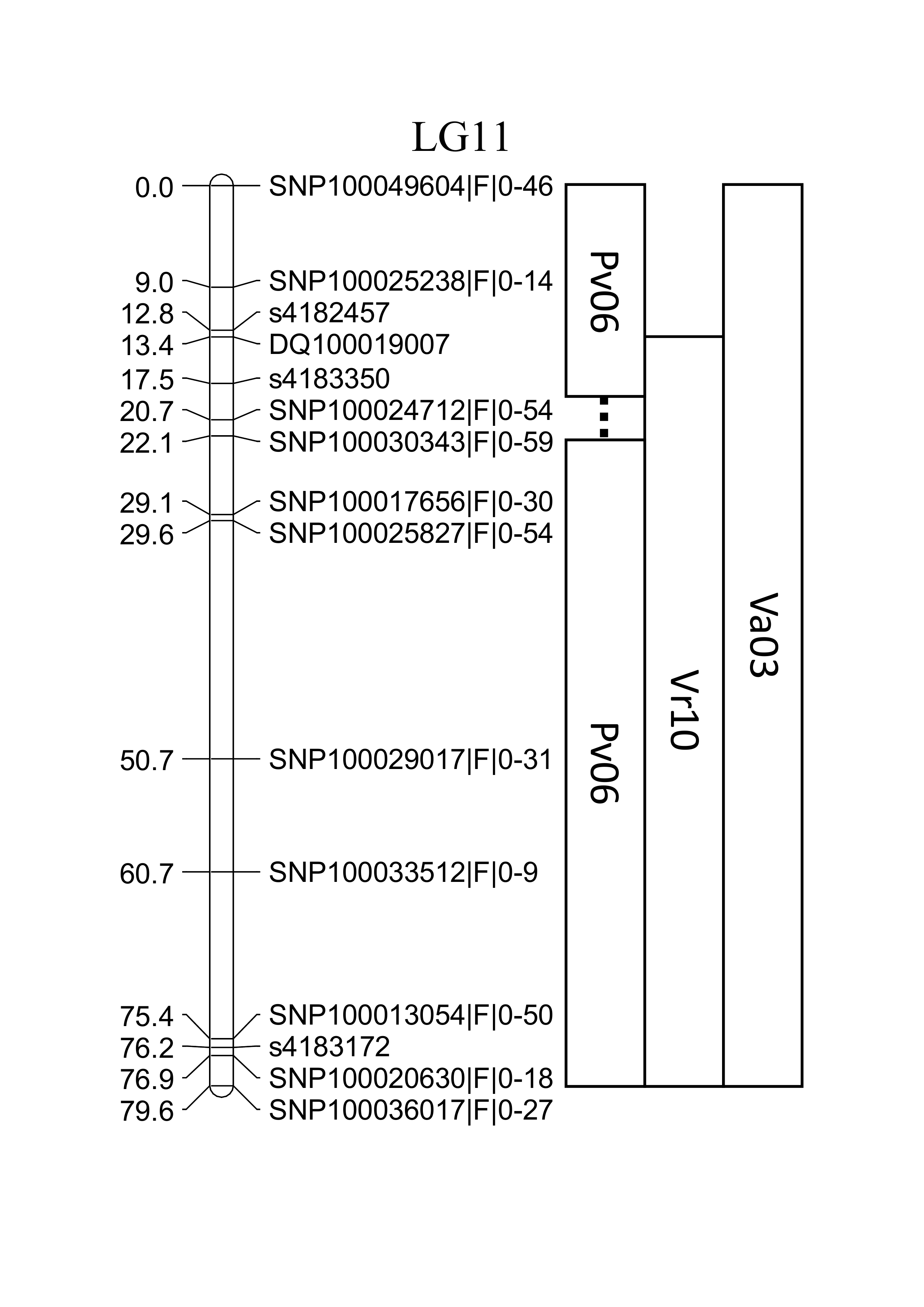

Supplement: Additional file 2: Figure S2a — and S2b. The syntenic relationship between linkage groups of TD population through (a) pre-selected common markers or (b) pre-selected common markers and 26% population-specific markers mappable to common bean genome (each line indicates one syntenic location data). The additional homologue information from the population-specific markers could help in further refining the target area underlying QTL. (ZIP 968kb) [file 12864_2016_3393_MOESM2_ESM.zip › 12864_2016_3393_MOESM2_ESM/Fig 4c/LG11.tif]

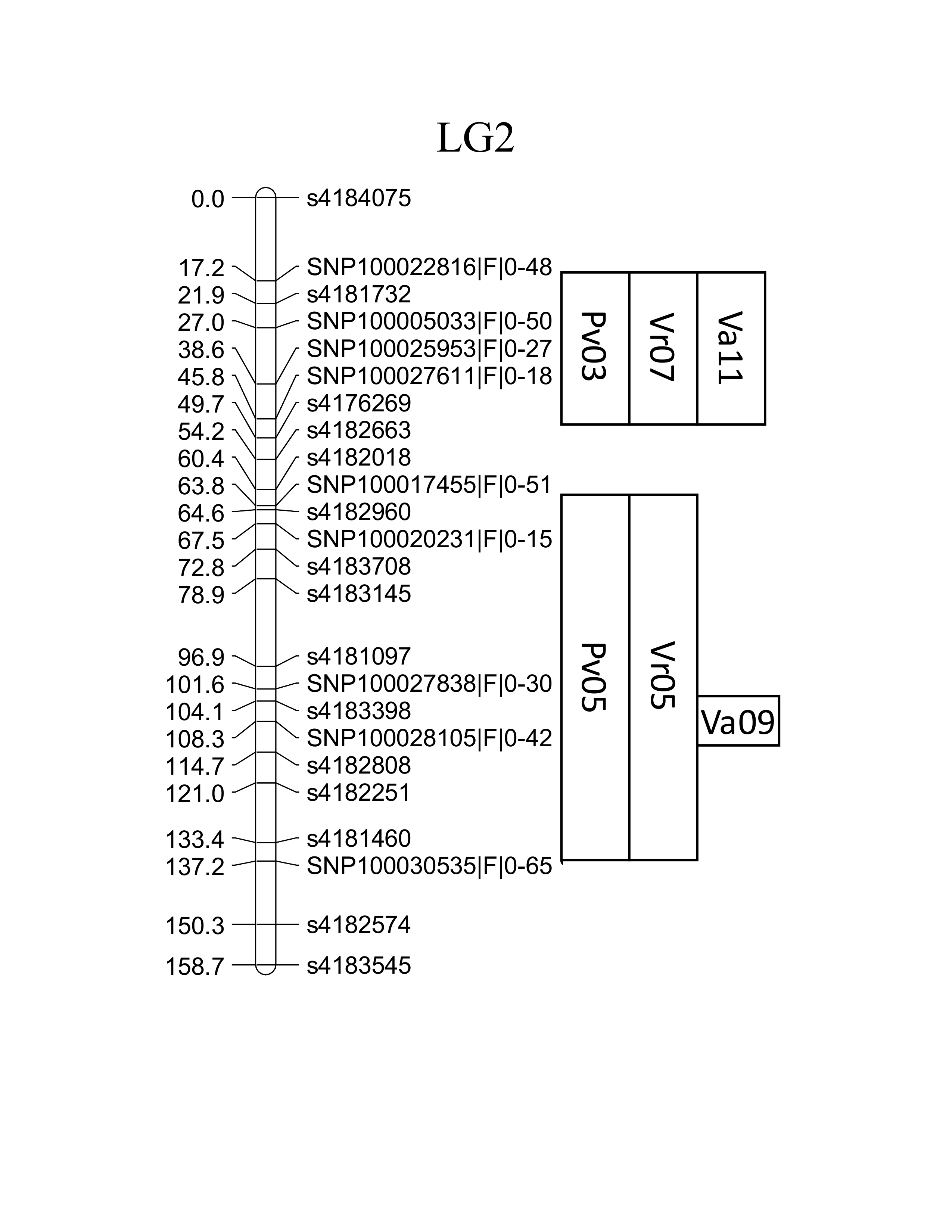

Supplement: Additional file 2: Figure S2a — and S2b. The syntenic relationship between linkage groups of TD population through (a) pre-selected common markers or (b) pre-selected common markers and 26% population-specific markers mappable to common bean genome (each line indicates one syntenic location data). The additional homologue information from the population-specific markers could help in further refining the target area underlying QTL. (ZIP 968kb) [file 12864_2016_3393_MOESM2_ESM.zip › 12864_2016_3393_MOESM2_ESM/Fig 4c/LG2.tif]

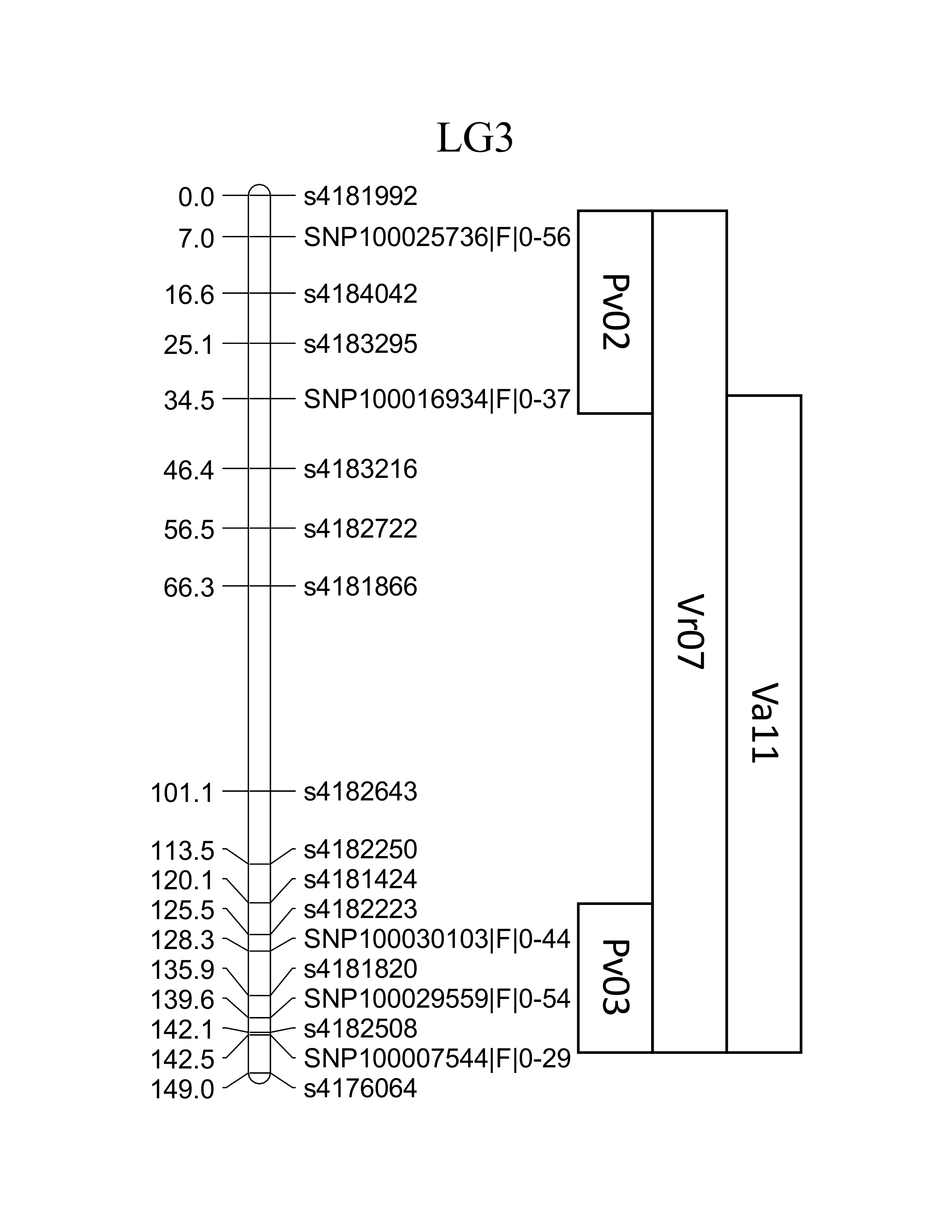

Supplement: Additional file 2: Figure S2a — and S2b. The syntenic relationship between linkage groups of TD population through (a) pre-selected common markers or (b) pre-selected common markers and 26% population-specific markers mappable to common bean genome (each line indicates one syntenic location data). The additional homologue information from the population-specific markers could help in further refining the target area underlying QTL. (ZIP 968kb) [file 12864_2016_3393_MOESM2_ESM.zip › 12864_2016_3393_MOESM2_ESM/Fig 4c/LG3.tif]

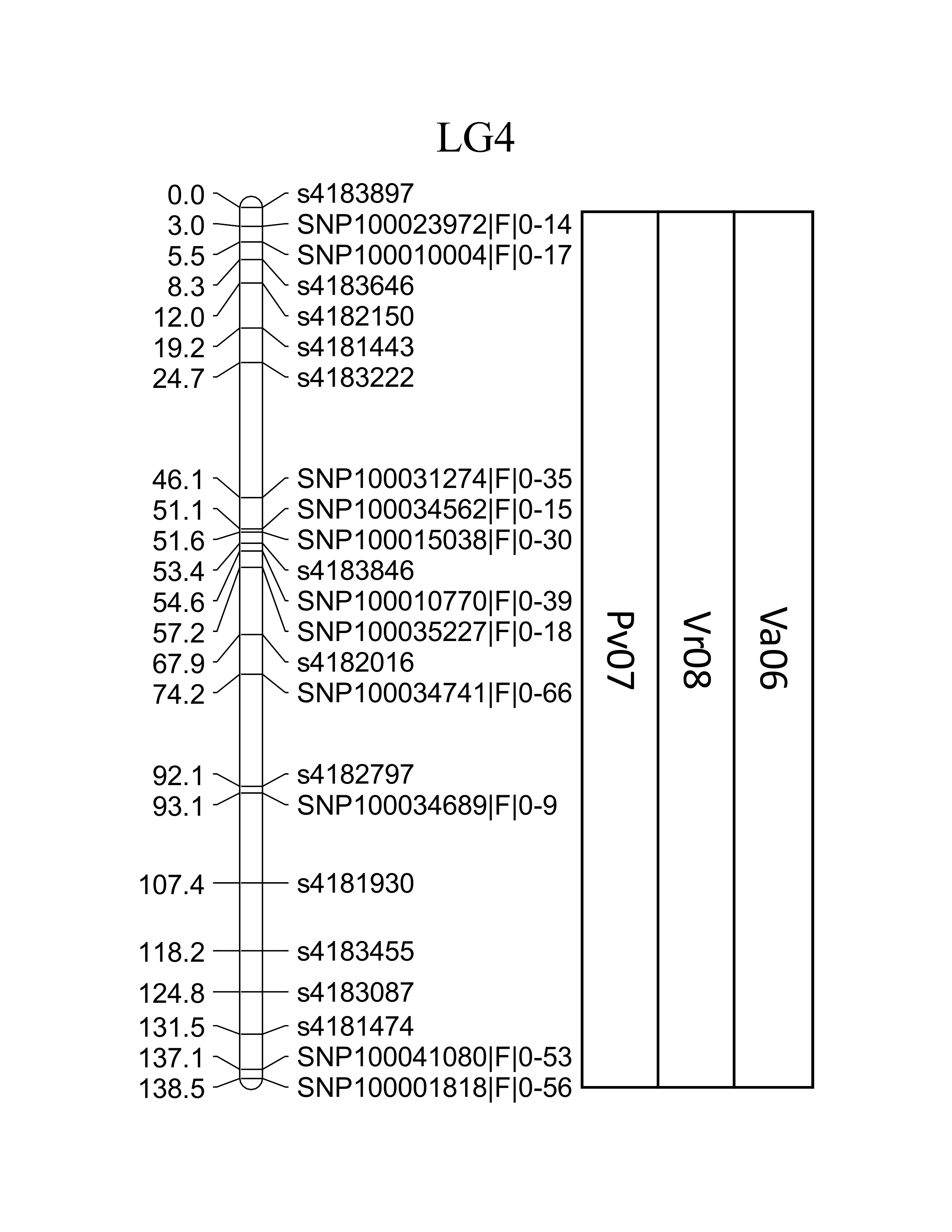

Supplement: Additional file 2: Figure S2a — and S2b. The syntenic relationship between linkage groups of TD population through (a) pre-selected common markers or (b) pre-selected common markers and 26% population-specific markers mappable to common bean genome (each line indicates one syntenic location data). The additional homologue information from the population-specific markers could help in further refining the target area underlying QTL. (ZIP 968kb) [file 12864_2016_3393_MOESM2_ESM.zip › 12864_2016_3393_MOESM2_ESM/Fig 4c/LG4.tif]

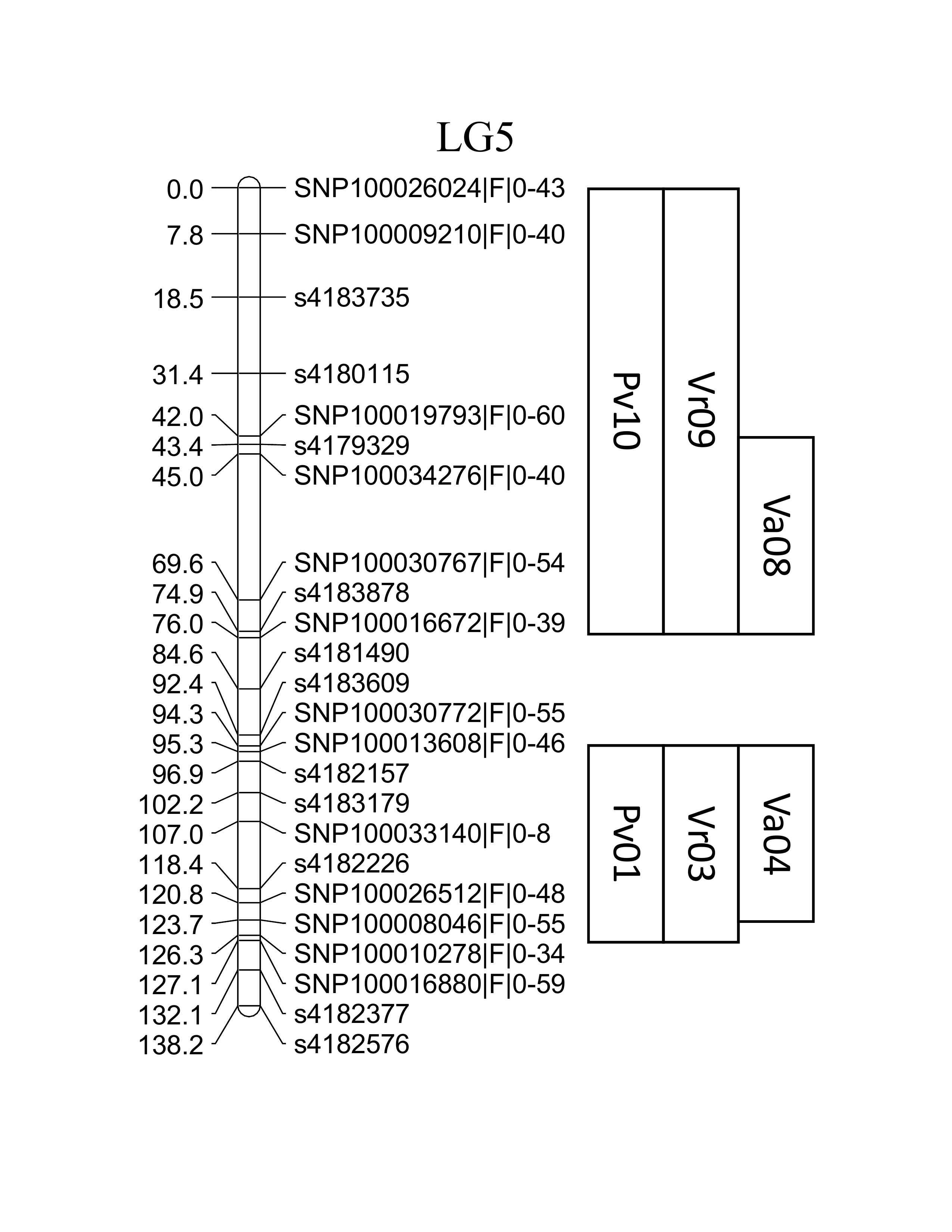

Supplement: Additional file 2: Figure S2a — and S2b. The syntenic relationship between linkage groups of TD population through (a) pre-selected common markers or (b) pre-selected common markers and 26% population-specific markers mappable to common bean genome (each line indicates one syntenic location data). The additional homologue information from the population-specific markers could help in further refining the target area underlying QTL. (ZIP 968kb) [file 12864_2016_3393_MOESM2_ESM.zip › 12864_2016_3393_MOESM2_ESM/Fig 4c/LG5.tif]

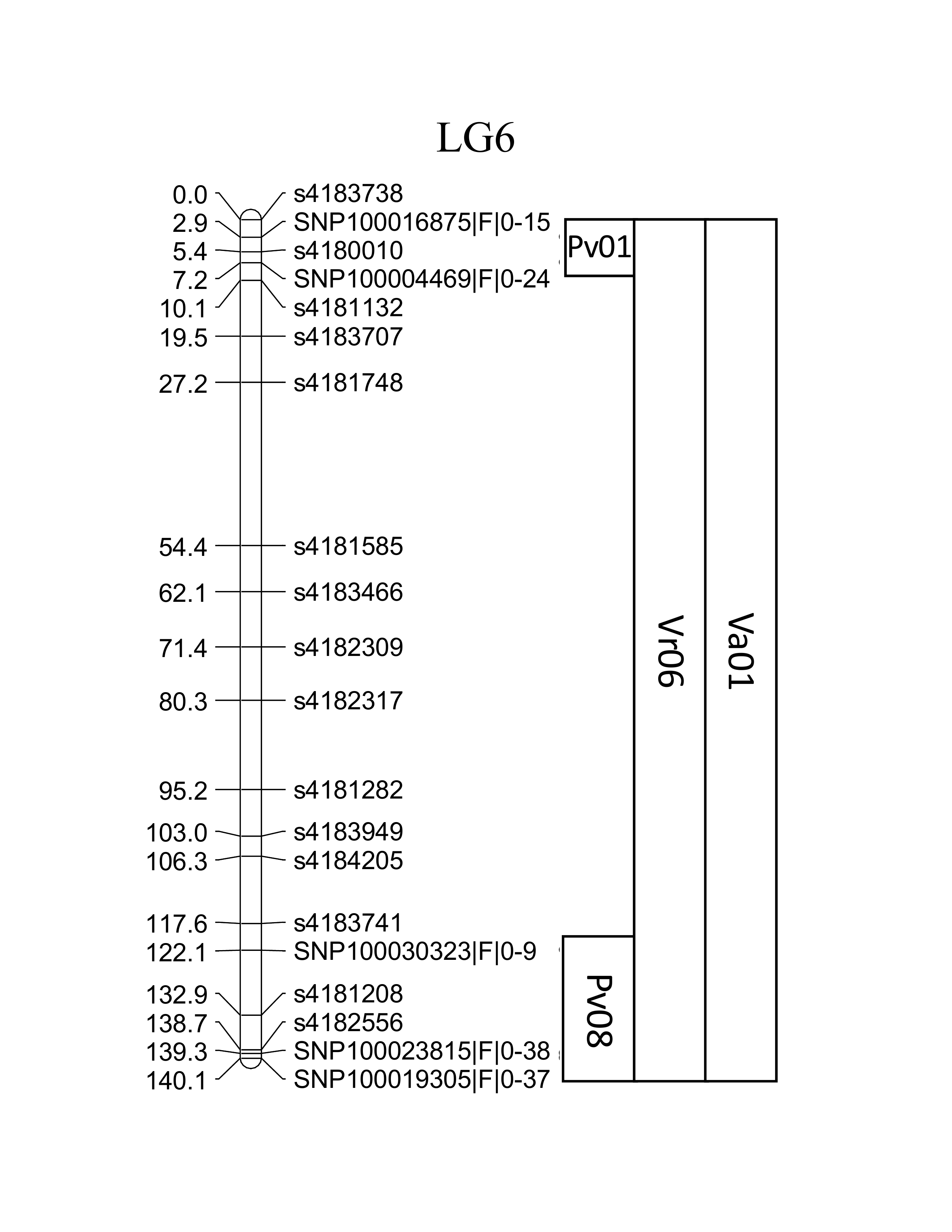

Supplement: Additional file 2: Figure S2a — and S2b. The syntenic relationship between linkage groups of TD population through (a) pre-selected common markers or (b) pre-selected common markers and 26% population-specific markers mappable to common bean genome (each line indicates one syntenic location data). The additional homologue information from the population-specific markers could help in further refining the target area underlying QTL. (ZIP 968kb) [file 12864_2016_3393_MOESM2_ESM.zip › 12864_2016_3393_MOESM2_ESM/Fig 4c/LG6.tif]

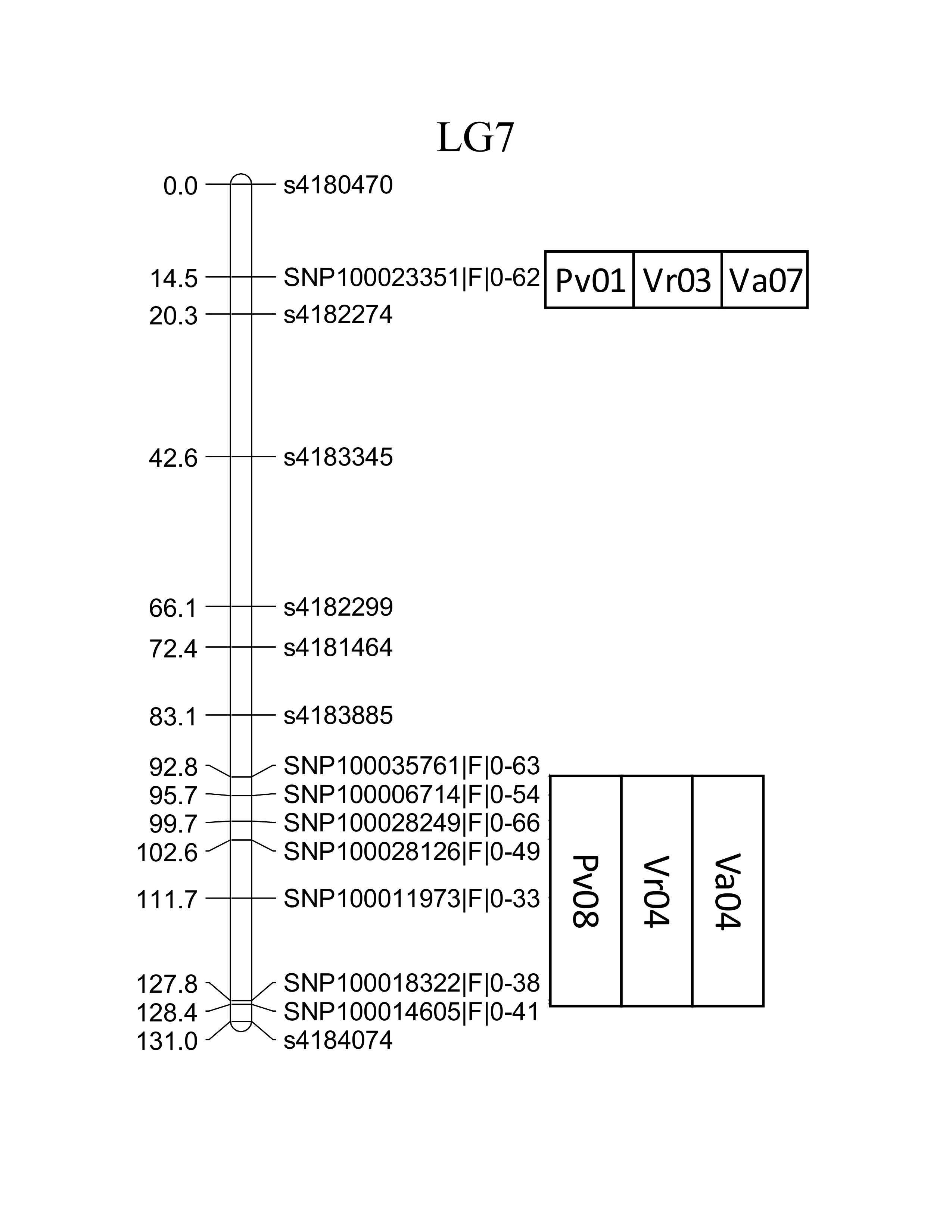

Supplement: Additional file 2: Figure S2a — and S2b. The syntenic relationship between linkage groups of TD population through (a) pre-selected common markers or (b) pre-selected common markers and 26% population-specific markers mappable to common bean genome (each line indicates one syntenic location data). The additional homologue information from the population-specific markers could help in further refining the target area underlying QTL. (ZIP 968kb) [file 12864_2016_3393_MOESM2_ESM.zip › 12864_2016_3393_MOESM2_ESM/Fig 4c/LG7.tif]

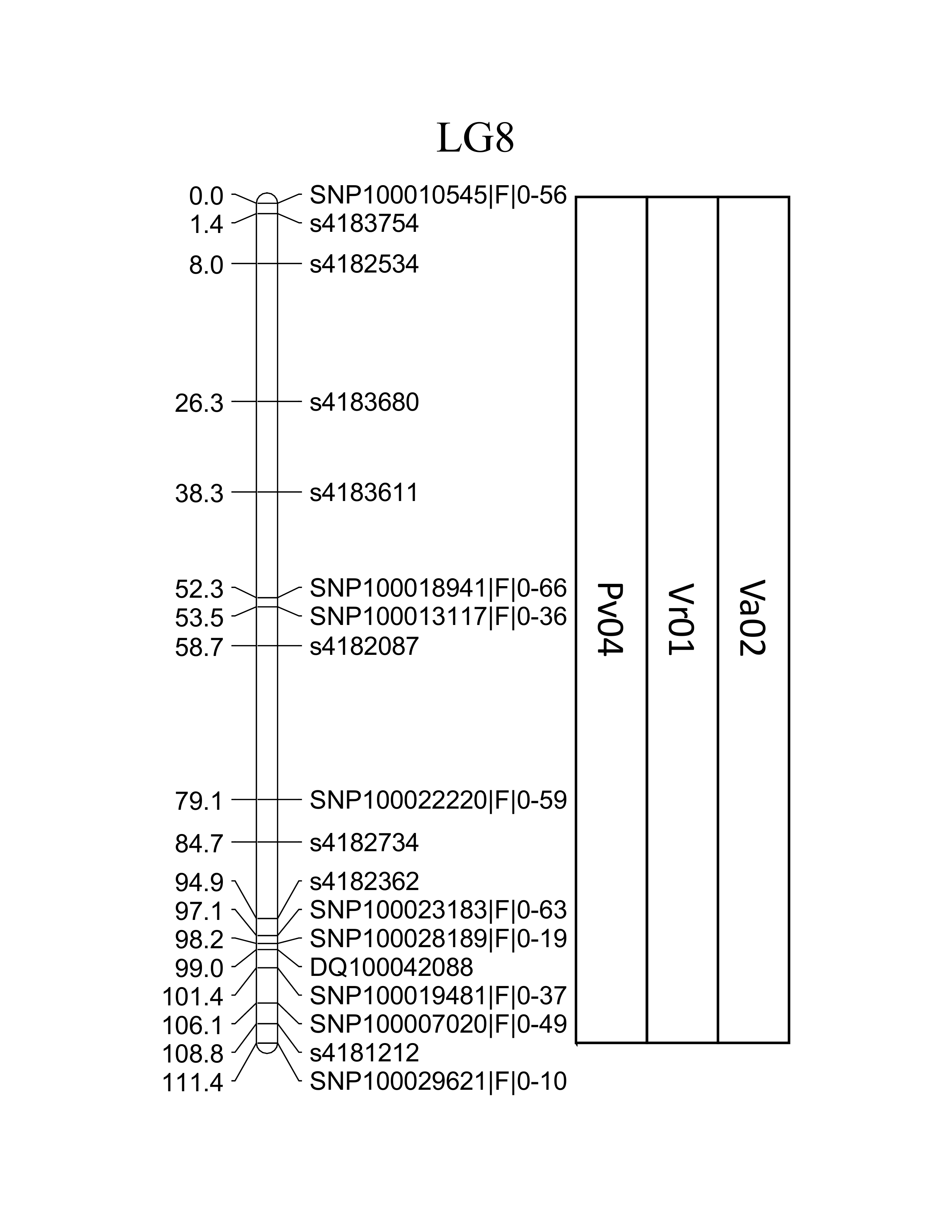

Supplement: Additional file 2: Figure S2a — and S2b. The syntenic relationship between linkage groups of TD population through (a) pre-selected common markers or (b) pre-selected common markers and 26% population-specific markers mappable to common bean genome (each line indicates one syntenic location data). The additional homologue information from the population-specific markers could help in further refining the target area underlying QTL. (ZIP 968kb) [file 12864_2016_3393_MOESM2_ESM.zip › 12864_2016_3393_MOESM2_ESM/Fig 4c/LG8.tif]

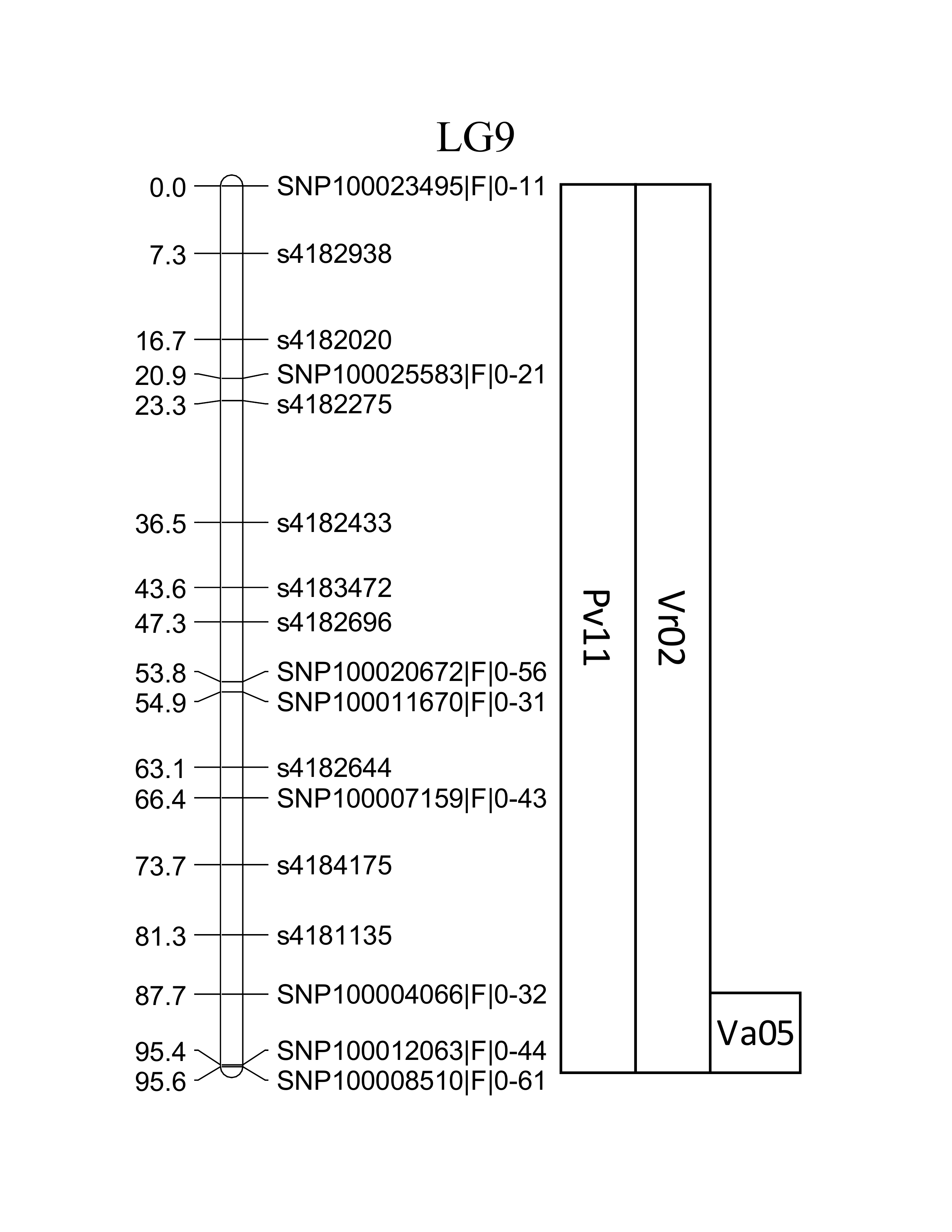

Supplement: Additional file 2: Figure S2a — and S2b. The syntenic relationship between linkage groups of TD population through (a) pre-selected common markers or (b) pre-selected common markers and 26% population-specific markers mappable to common bean genome (each line indicates one syntenic location data). The additional homologue information from the population-specific markers could help in further refining the target area underlying QTL. (ZIP 968kb) [file 12864_2016_3393_MOESM2_ESM.zip › 12864_2016_3393_MOESM2_ESM/Fig 4c/LG9.tif]
